# Favourable Perceptions of Electronic Cigarettes Relative to Cigarettes and the Associations with Susceptibility to Electronic Cigarette Use in Hong Kong Chinese Adolescents

**DOI:** 10.3390/ijerph15010054

**Published:** 2018-01-01

**Authors:** Lok Tung Leung, Sai Yin Ho, Jianjiu Chen, Man Ping Wang, Tai Hing Lam

**Affiliations:** 1 School of Public Health, The University of Hong Kong, Hong Kong, China; lloktung@hku.hk (L.T.L.); jjchen@hku.hk (J.C.); hrmrlth@hku.hk (T.H.L.); 2 School of Nursing, The University of Hong Kong, Hong Kong, China; mpwang@hku.hk

**Keywords:** electronic cigarettes, perceptions, adolescents, Chinese

## Abstract

We investigated favourable perceptions of electronic cigarettes (ECs) relative to cigarettes and their associations with EC use susceptibility in adolescents. Hong Kong Chinese Secondary 1–6 (U.S. grade 7–12) students (*n* = 40,202) were surveyed in 2014/2015 on EC use, cigarette smoking, favourable perceptions of ECs relative to cigarettes, EC use susceptibility, family smoking, and socio-demographic characteristics. Cox regression yielded adjusted prevalence ratios (APRs) of EC use susceptibility in never users, excluding those unaware of ECs. In all students, 8.9% were ever EC users, 47.2% reported favourable perceptions of ECs relative to cigarettes, such as less likely to cause accidents (25.2%) and less harmful to users (24.5%), and 28.9% did not know ECs. Among never EC users who were aware of ECs (*n* = 24,663), EC use susceptibility was associated with each of the favourable perceptions, especially greater attractiveness (APR 2.84, 95% CI 2.53–3.19), and better parental (2.75, 2.41–3.15) and school acceptability (2.56, 2.15–3.05). An increased number of favourable perceptions of ECs relative to cigarettes was associated more strongly with the susceptibility (*p* for trend < 0.001). Our findings inform strategies to reduce unwarranted favourable perceptions and prevent adolescent EC use.

## 1. Introduction

Electronic cigarettes (ECs) are purported to be a smoking cessation aid and a safer alternative to conventional cigarettes. Some studies have found an increased cessation rate in EC users [1,2,3,4,5], but scant high-quality evidence and contradictory results in other studies warrant further evaluation of their effectiveness [6,7]. With ECs appearing less harmful than cigarettes [8,9], substitution of cigarettes with ECs is expected to bring positive public health impacts [10]. However, the long-term harms are yet unknown [11]. Even in the short term, users are at risks of explosion injuries and exposure to nicotine and harmful chemicals [11,12].

Emerging studies have found smoking initiation and heavier smoking subsequent to EC use in young people [7,13,14,15,16,17], which are the major concern about EC use. The prevalence of past 30-day or at-least-monthly EC use in adolescents was 29.9% in Poland [18], 11.3% in the United States (U.S.) [11], 4.7% in Korea [19], 3.2% in Ireland [20], and 1.7% in the United Kingdom (UK) [21]. Curiosity and appealing flavours are the most popular reasons for use across smoking status [22,23]. Adolescent smokers who use ECs are less likely to abstain from smoking [24], while more frequent EC use predicts heavier smoking [14]. Similarly, EC use in never smokers prospectively predicts desensitisation to the harms of smoking [16], and smoking susceptibility and initiation [13,15,25]. Such links between EC use and cigarette smoking were also observed in Hong Kong Chinese adolescents, despite their relatively low prevalence of EC use (past 30 day use 1.1% in 2012/2013) [26]. We also observed an association between adolescent EC use and respiratory symptoms in a previous study [27].

The scientific community remains split on the EC issue. The complicated situation is reflected in EC-related policies across countries. The UK, for example, approves the use of ECs as a cessation tool on prescription but bans selling ECs to minors [28]. Some countries (e.g., Italy, the U.S., and Korea) allow selling ECs to adults and regulate the product contents or packaging, while some (e.g., Greece, Singapore, and Turkey) completely ban its sale [29].

In Hong Kong, nicotine-containing ECs must register with the government before sale or distribution, but none have registered [30,31]. Legislation to outlaw the sale, distribution, and promotion of all ECs is still under consideration. Currently, ECs that claim to be nicotine-free are readily available in retail outlets frequented by adolescents, and nicotine-containing ECs are easily accessible online [26,32].

Perceptions can affect health behaviours, including smoking [33]. Understanding adolescent perceptions of ECs may inform strategies to prevent EC use. ECs allow users to imitate cigarette smoking, and are marketed as a trendy and healthier product with diverse flavours [34,35]. Compared with cigarettes, adolescents often find ECs more appealing, and consider them less harmful, less addictive, safer, cleaner, more socially acceptable, trendier, easier to access, and easier to hide [21,36,37,38,39]. The perception that ECs are less harmful than cigarettes predicts EC use in adolescents [40,41]. Other favourable perceptions of ECs relative to cigarettes may also predict EC use, but have rarely been studied. We therefore examined to what degree adolescents had favourable perceptions of ECs compared with cigarettes, using data from a territory-wide survey in secondary school students in Hong Kong. Associations between the perceptions and EC use susceptibility in never EC users, a strong predictor of future EC use [42], were also examined. As EC use may promote smoking in non-smokers, we did subgroup analyses on the associations in never smokers and ex-smokers. Age and sex-specific associations were also analysed.

## 2. Materials and Methods 

Secondary 1 to 6 (U.S. grade 7–12) students (95% response rate) of 92 schools randomly selected from all the 18 districts in Hong Kong were invited to complete an anonymous questionnaire adapted from the Global Youth Tobacco Survey in 2014/2015 [43]. An invitation letter for the voluntary survey was sent to parents via students. Parents refusing to consent could ask their children to return a blank answer sheet during the survey, and students could decline participation even with parental consent. The survey was conducted in classrooms, and completed answer sheets were collected and sealed in an opaque envelope by research staff in front of students. Ethical approval was granted by the Institutional Review Board of the University of Hong Kong/Hospital Authority Hong Kong West Cluster (ethical code: UW 14-487).

Cigarette smoking status was assessed by 2 questions. Students were first asked “Which statement best describes your cigarette smoking status?” with response options “I have never smoked”, “I smoked once or a few times”, “I used to smoke (not every day), but have quit now”, “I used to smoke every day, but have quit now”, “I smoke on some days now”, and “I smoke every day now”. They were then asked “How many days did you smoke in the past 30 days?” with response options “0 day”, “1–2 days”, “3–5 days”, “6–9 days”, “10–19 days”, “20–29 days”, and “30 days” [44]. Those reporting never smoking and no smoking in the past 30 days were defined as never smokers, while those who had quit smoking and did not smoke in the past 30 days were defined as ex-smokers. Hereafter, the terms of smokers, never smokers and ex-smokers, refer only to cigarette smoking.

EC use status was assessed with similar questions, “Which statement best describes your EC use status?” and “How many days did you use ECs in the past 30 days?”, and similar response options. Never EC users referred to students who reported never EC use and did not use ECs in the past 30 days. Adapted from the assessment of susceptibility to cigarette smoking [26,45], “Do you think you will use an EC in the next 12 months?” and “Will you use an EC if one of your good friends offers you one?” assessed susceptibility to EC use. Response options to each question included “definitely not”, “probably not”, “probably yes”, and “definitely yes”. Students answering “definitely not” to both questions were defined as not susceptible to EC use, and otherwise as susceptible.

For favourable perceptions of ECs relative to cigarettes, students were asked “Compared with cigarettes, do you think ECs are:” with a checklist of 13 potential advantages of ECs over cigarettes, “none of the above” and “don’t know ECs”. The potential advantages included “less harmful to users”, “more attractive”, “easier to use at home unnoticed”, and “EC use in children is better accepted by parents”, etc. They were derived with reference to the reasons for and perceptions of EC use discussed in other studies [22,23,36,37,38,39]. Students could select one or more of the potential advantages, “none of the above”, or “do not know ECs”. Other information collected included age, sex, perceived family affluence (relatively poor/poor to average/average/average to rich/relatively rich), highest parental education (primary or below/secondary/tertiary/unknown), and family smoking (no/yes).

A total of 40,202 students remained in the database after excluding 833 (2.0%) with missing information on age, sex or grade, or over 50% missing data. STATA 13.0 (StataCorp LLC, College Station, TX, USA) was used for analysis. Descriptive data were weighted by official age, sex, and grade distribution of Hong Kong students in 2014/2015 provided by the Education Bureau. Favourable perceptions of ECs relative to cigarettes were analysed individually and as a combined variable, the number of favourable perceptions. Prevalence of the favourable perceptions relative to cigarettes was calculated in all students, and the associations with susceptibility to EC use were analysed in never EC users. Since EC use susceptibility was not rare in our sample (>10%), odds ratios yielded from logistic regression may overestimate the associations; prevalence ratios (PRs, ratio of 2 prevalences) were calculated using Cox regression with constant time at risk and robust variance estimators [46,47]. Age, sex, perceived family affluence, highest parental education, cigarette smoking status, family smoking, and school clustering effect were adjusted for. The analysis was repeated in never users who had never smoked and formerly smoked, adjusting for the same covariates except smoking status. Age and sex-specific associations were also analysed by dividing the never users by age (under 15 years old vs. 15 years old or above) and sex, respectively. To examine differences in the associations by age and sex, interactions between age and sex and favourable perceptions were tested in the adjusted models. Students unaware of ECs were excluded from all regression analyses. A sensitivity analysis was conducted, with EC use susceptibility defined as “definitely yes” or “probably yes” in response to either susceptibility questions.

## 3. Results

Among all students (*n* = 40,202), 51.5% were boys and the mean age was 14.9 (standard deviation 1.8) years. Table 1 shows that 29.4% perceived their family affluence as below average, 78.4% had parents with at least secondary education, and 34.9% lived with smokers. The prevalence of ever cigarette smoking was 12.7% (95% CI 12.3–13.0%), including 1.7% (95% CI 1.6–1.9%) ex-smokers, and ever EC use was 8.9% (95% CI 8.6–9.2%).

Many students (47.2%) had at least 1 favourable perception of ECs relative to cigarettes, including 24.1% having 1–2, 13.6% having 3–4, and 9.5% having 5 or more favourable perceptions, while less than one-third (28.9%) did not know ECs (Table 2). The prevalence of individual favourable perceptions ranged from about 1% to 25%. Students most commonly perceived that ECs were less likely to cause accidents such as fires and burns (25.2%), were less harmful to users (24.5%), and were less harmful to others (22.6%) than cigarettes.

EC use susceptibility was identified in 16.7% of all students (Table 1). Among never EC users who were aware of ECs (*n* = 24,663), 12.2% of all were susceptible (Table 3). Susceptibility was more common in the ex-smoker subgroup (34.5%) than never smoker subgroup (10.1%) (*p* for chi-square < 0.001). Each favourable perception of ECs relative to cigarettes was significantly associated with EC use susceptibility in both crude and adjusted models. The strongest associations were observed for perceiving ECs as more attractive (adjusted PR [APR] 2.84, 95% CI 2.53–3.19), and EC use in children as better accepted by parents (2.75, 2.41–3.15) and schools (2.56, 2.15–3.05). Increased number of favourable perceptions was associated with higher APRs (*p* for trend < 0.001), compared with none. This indicates that never users that had more favourable perceptions of ECs relative to cigarettes were more likely to be susceptible. Subgroup analysis showed similar patterns of associations in those who had never smoked. The associations were weaker in ex-smokers, and were only significant for perceiving ECs as less likely to cause accidents (1.44, 1.07–1.93), less harmful to users (1.52, 1.11–2.07), less harmful to others (1.65, 1.27–2.14), more attractive (1.45, 1.00–2.11), more environmentally friendly (1.50, 1.18–1.92), and more convenient (1.43, 1.05–1.96). Having more favourable perceptions of ECs had a stronger association with susceptibility to EC use (*p* for trend = 0.004). Sensitivity analysis based on an alternative definition of susceptibility to EC use yielded similar results for never EC users and the 2 subgroups.

The prevalence of EC use susceptibility was similar in younger and older students (12.4% vs. 12.1%), and slightly higher in girls than boys (12.8% vs. 11.7%, *p* for chi-square < 0.05) (Table 4 and Table 5). All the favourable perceptions were significantly associated with susceptibility in all subgroups, with stronger associations for having more favourable perceptions. The associations were significantly stronger in younger than older students for several favourable perception, such as perceiving ECs as less harmful to users (*p* for interaction < 0.001) and others (*p* for interaction < 0.01), and perceiving ECs as easier for minors to buy (*p* for interaction < 0.001). Girls were more likely than boys to be susceptible to EC use for perceiving ECs as more attractive (*p* for interaction < 0.05) and EC use in children as better accepted by parents (*p* for interaction < 0.01).

## 4. Discussion

We reported favourable perceptions of ECs relative to cigarettes in Hong Kong Chinese adolescents. To our knowledge, this is the first study to examine various perceptions in relation to susceptibility to EC use in never EC users, while previous studies have predominantly focused on relative harm perceptions and differences in perceptions by EC use and smoking status [39,48,49]. In Hong Kong, about 70% of adolescents were aware of ECs and 9% had ever used ECs. Despite the low EC use prevalence, one-sixth of adolescents were susceptible to EC use, and nearly half perceived any advantages of ECs over cigarettes, including 9.5% who perceived at least 5 advantages. Although studies have suggested potential benefits of ECs [1,9], ECs are not harmless and the long-term risks of use are still unclear [11]. For non-smokers in particular, the great majority of adolescents in society, the potential benefits of switching from cigarettes to ECs do not apply, and ECs may be a gateway to smoking [13,25,41,50]. Preventive measures are needed, and it is worthwhile to explore risk factors of EC use susceptibility in adolescents, which prospectively predicts EC use initiation [42], to inform prevention work.

We found that adolescent never users who were aware of ECs were more likely to be susceptible to EC use for having any of the favourable perceptions examined, including ECs being less harmful, easier to buy, and easier to use unnoticed than cigarettes, which had been reported as reasons for EC use by adolescents in previous studies [23,37].

Harm perceptions predict substance use in adolescents [33,51]. Sustained anti-smoking campaigns in Hong Kong have educated the public about the harms of cigarettes [52], while the potential health hazards of the novel product, ECs, might not be well recognised. Never users who considered ECs less harmful than cigarettes were more likely to be susceptible, which is consistent with prior research showing a positive association between such belief and ever EC use [40,41]. The findings suggest that adolescents tend to use products that seem less risky. The associations remain robust in never smokers and ex-smokers, suggesting that adolescents who are deterred by the harms of cigarettes may be attracted to use ECs by messages implying lower harmfulness of ECs.

Perceived greater attractiveness and better acceptability of ECs than cigarettes, although not common, are worthy of notice for their particularly robust associations with EC use susceptibility. Cigarettes in Hong Kong are mandatory to have health warnings on packages, and are available in only limited flavours. On the contrary, ECs lack warnings, have stylish designs and diverse flavours, and allow users to perform vape tricks [35]. These properties have been linked to youth appeal [23,53], and might make ECs more attractive than cigarettes and arouse adolescent interest in trying ECs. Never smokers seemed more susceptible to EC use than ex-smokers for perceiving ECs as more attractive than cigarettes. Future studies should identify the attributes of ECs that attract adolescents, especially never smokers.

In line with the inverse association of perceived parental and school disapproval with adolescent smoking [54,55,56], we found that perceived better parental and school acceptability of EC use than smoking was linked to EC use susceptibility. The associations remained strong and significant in never smokers but not ex-smokers. These perceptions may not matter much to ex-smokers, as they have already tried cigarettes, which are clearly disapproved of by schools and most parents. Nonetheless, parents and schools should hold equally negative attitudes towards ECs and cigarettes to discourage EC use and smoking at least in never-smoking adolescents.

Never EC users that had more favourable perceptions relative to cigarettes were more likely to be susceptible to EC use. Some of the perceptions, such as that ECs are cleaner than cigarettes, are inherent merits of ECs over cigarettes. However, unwarranted perceptions (e.g., ECs are easier for minors to buy) should be reduced to help reduce the susceptibility. Our findings suggest restrictions on product designs, access restrictions, education campaigns, and school policies. Attributes of ECs that may appeal to adolescents, such as stylish designs and flavour variability, should be removed. Movie stars and cartoons popular among adolescents are sometimes featured on ECs on top of the stylish designs [35]. ECs in candy, fruit, and menthol flavours are regarded as less harmful by adolescents and appeal to them more [57]. To reduce the youth appeal of ECs, the product designs should be vigorously regulated, while the number of flavours should be limited and flavours particularly appealing to adolescents should be prohibited.

Never users, especially those younger than age 15, who perceived easier access to ECs than cigarettes, were more likely to be susceptible to EC use. Perceived accessibility to cigarettes dose-dependently predicted smoking initiation in adolescents [58]. In Hong Kong, selling cigarettes but not ECs to minors is prohibited. While cigarettes are typically sold in newspaper stands, convenience stores, and supermarkets, ECs are sold online and among other trendy products in retail stores frequented by adolescents. Age restriction on EC purchase and point-of-sale restriction should be imposed to curb youth access to ECs and reduce the perceived accessibility.

Our findings suggest the need to raise awareness of the potential harms of ECs in adolescents. Given the rapidly growing research on ECs, up-to-date evidence of the benefits and harms of ECs should be constantly reviewed to inform education campaigns. Unfounded health and cessation claims regarding ECs should be counteracted [59]. Besides, the campaigns should be supported by school policies that involve parents and strongly disapprove of student EC use and cigarette smoking. School policies are more successful in preventing health-risk behaviours in students if parents are involved through ways such as participating in school health activities and receiving health education materials from schools [60]. Parent engagement may help extend the anti-EC education from schools to homes and increase the likelihood that adolescents consistently perceive the disapproval of EC use and smoking from parents, as well as schools. The stronger association between perceiving ECs as less harmful than cigarettes and EC use susceptibility found in younger than older adolescents implies the need for more intensive education at younger age.

This study has some limitations. First, all data were self-reported and are subject to reporting bias. The assessment of smoking status has been validated in Chinese adolescents [61], but that of EC use status has not. Measures, such as masking the meanings of response options with multiple-choice answer sheets and sealing completed answer sheets in front of students, were taken to ensure confidentiality of the anonymous survey and encourage candid reporting. Besides, the assessment of susceptibility to EC use was adapted from the validated questions measuring susceptibility to cigarette smoking in adolescents, including the intention in the next 12 months and when a good friend offered a cigarette [45]. Validity of the assessment was supported by the robust association between susceptibility to EC use and ever EC use in the present sample (Table S1 in the Supplementary Materials). A similar assessment compositely measuring susceptibility to EC use in the future and when a good friend offered an EC has been shown to be an independent prospective predictor of EC use in adolescent never users [42], also lending support to our assessment. Second, causality cannot be inferred based on our cross-sectional observational data. While adolescents that had favourable perceptions of ECs relative to cigarettes had a higher likelihood of EC use susceptibility, it is also plausible that susceptible adolescents tended to have more favourable perceptions of ECs. Third, although potential confounders were adjusted for in the regression analyses, residual confounding cannot be ruled out.

## 5. Conclusions

Many Hong Kong adolescents had favourable perceptions of ECs relative to cigarettes, especially that ECs were less likely to cause accidents and cause less harm to users. In never EC users, perceived greater attractiveness of ECs and better parental and school acceptability of EC use were the strongest predictors of susceptibility to EC use among the favourable perceptions. Those having an increased number of favourable perceptions were more likely to be susceptible. Given the risks of adverse events of EC use, measures are needed to reduce unwarranted favourable perceptions of ECs relative to cigarettes and prevent EC use initiation in adolescents.

## Figures and Tables

**Table 1:** Background characteristics (*n* = 40,202).

Characteristics	% ^1^
**Age (mean ± SD), years**	14.9 ± 1.8
**Sex**	
Boys	51.5
Girls	48.5
**Perceived family affluence**	
Relatively poor	6.1
Poor to average	23.3
Average	55.0
Average to Rich	13.2
Relatively rich	2.4
**Highest parental education**	
Primary or below	6.0
Secondary	51.7
Tertiary	26.7
Unknown	15.5
**Family smoking**	
No	65.1
Yes	34.9
**EC use status**	
Never	91.2
Ever	8.9
**Cigarette smoking status**	
Never	87.3
Ever	12.7
Ex-smokers	1.7
**EC use susceptibility**	
Not susceptible	83.3
Susceptible	16.7

^1^ Percentages were weighted by age, sex and grade.

**Table 2:** Favourable perceptions of ECs relative to cigarettes (*n* = 40,202).

Favourable Perceptions of ECs Relative to Cigarettes	% ^1^
Less likely to cause accidents such as fires and burns	25.2
Less harmful to users	24.5
Less harmful to others	22.6
More attractive	5.3
More chic	4.8
Easier for minors to buy	9.8
Easier to use at home unnoticed	4.5
Easier to use at school unnoticed	3.8
More environmentally friendly	12.4
More convenient	11.2
Cleaner	16.7
EC use in children is better accepted by parents	1.6
EC use in students is better accepted by schools	1.3
**Number of favourable perceptions**	
0	23.9
1–2	24.1
3–4	13.6
5–13	9.5
Did not know ECs	28.9

^1^ Percentages were weighted by age, sex and grade.

**Table 3:** Association between favourable perceptions of ECs relative to cigarettes and susceptibility in never EC users.

	Never EC Users ^1^					
	All (*n* = 24,663, EC Use Susceptibility = 12.2%)		Never Smokers (*n* = 22,412, EC Use Susceptibility = 10.1%)		Ex-Smokers (*n* = 316, EC Use Susceptibility = 34.5%)	
Favourable Perceptions of ECs Relative to Cigarettes ^2^	Crude PR (95% CI)	Adjusted PR ^3^ (95% CI)	Crude PR (95% CI)	Adjusted PR ^4^ (95% CI)	Crude OR (95% CI)	Adjusted PR ^4^ (95% CI)
Less likely to cause accidents such as fires and burns	1.77 (1.65–1.90) ***	1.69 (1.58–1.80) ***	1.89 (1.74–2.05) ***	1.85 (1.71–2.00) ***	1.41 (0.97–2.05)	1.44 (1.07–1.93) *
Less harmful to users	2.44 (2.27–2.62) ***	2.26 (2.09–2.45) ***	2.56 (2.36–2.78) ***	2.56 (2.33–2.80) ***	1.48 (1.03–2.15) *	1.52 (1.11–2.07) **
Less harmful to others	2.25 (2.09–2.41) ***	2.09 (1.91–2.30) ***	2.31 (2.13–2.50) ***	2.29 (2.04–2.57) ***	1.58 (1.09–2.30) *	1.65 (1.27–2.14) ***
More attractive	3.28 (2.99–3.60) ***	2.84 (2.53–3.19) ***	3.65 (3.28–4.07) ***	3.58 (3.13–4.10) ***	1.41 (0.85–2.34)	1.45 (1.00–2.11) *
More chic	2.54 (2.29–2.81) ***	2.25 (2.01–2.51) ***	2.87 (2.55–3.23) ***	2.84 (2.53–3.18) ***	0.80 (0.37–1.71)	0.81 (0.43–1.49)
Easier for minors to buy	2.51 (2.31–2.72) ***	2.30 (2.11–2.52) ***	2.75 (2.51–3.02) ***	2.73 (2.47–3.02) ***	1.09 (0.67–1.78)	1.07 (0.64–1.79)
Easier to use at home unnoticed	2.43 (2.17–2.72) ***	2.08 (1.86–2.33) ***	2.53 (2.22–2.89) ***	2.43 (2.14–2.75) ***	1.21 (0.66–2.20)	1.20 (0.75–1.93)
Easier to use at school unnoticed	2.27 (2.01–2.57) ***	2.03 (1.79–2.29) ***	2.32 (2.01–2.68) ***	2.28 (2.01–2.59) ***	0.92 (0.43–1.97)	0.95 (0.50–1.81)
More environmentally friendly	2.10 (1.95–2.28) ***	1.95 (1.78–2.15) ***	2.16 (1.97–2.36) ***	2.15 (1.91–2.42) ***	1.53 (1.01–2.31) *	1.50 (1.18–1.92) **
More convenient	2.15 (1.99–2.34) ***	1.94 (1.78–2.11) ***	2.17 (1.98–2.39) ***	2.16 (1.95–2.40) ***	1.45 (0.96–2.18)	1.43 (1.05–1.96) *
Cleaner	2.15 (2.00–2.31) ***	2.04 (1.87–2.23) ***	2.25 (2.07–2.45) ***	2.25 (2.02–2.50) ***	1.20 (0.80–1.80)	1.17 (0.83–1.65)
EC use in children is better accepted by parents	3.25 (2.77–3.81) ***	2.75 (2.41–3.15) ***	3.65 (3.04–4.38) ***	3.50 (3.01–4.08) ***	1.04 (0.33–3.27)	1.03 (0.33–3.24)
EC use in students is better accepted by schools	3.07 (2.54–3.71) ***	2.56 (2.15–3.05) ***	3.34 (2.68–4.16) ***	3.29 (2.72–3.98) ***	1.01 (0.37–2.73)	0.92 (0.43–1.97)
**Number of favourable perceptions**						
0	1	1	1	1	1	1
1–2	2.27 (2.03–2.55) ***	2.22 (1.97–2.51) ***	2.43 (2.14–2.77) ***	2.52 (2.23–2.86) ***	1.45 (0.84–2.51)	1.55 (1.03–2.34) *
3–4	3.67 (3.27–4.12) ***	3.43 (2.97–3.96) ***	3.85 (3.37–4.40) ***	3.91 (3.35–4.57) ***	2.20 (1.27–3.80) **	2.30 (1.55–3.42) ***
5–13	6.08 (5.42–6.81) ***	5.45 (4.72–6.30) ***	6.81 (5.97–7.77) ***	6.91 (5.94–8.03) ***	2.10 (1.14–3.87) *	2.18 (1.38–3.46) **
*p* for trend	<0.001	<0.001	<0.001	<0.001	0.025	0.004

^1^ Students not knowing ECs were excluded. ^2^ The reference group for each favourable perception comprised students who did not select the corresponding perception. ^3^ Adjusted for age, sex, perceived family affluence, highest parental education, ever smoking status, family smoking, and school clustering effect. ^4^ Adjusted for age, sex, perceived family affluence, highest parental education, family smoking, and school clustering effect. * *p* < 0.05, ** *p* < 0.01, *** *p* < 0.001.

**Table 4:** Association between favourable perceptions of ECs relative to cigarettes and susceptibility in never EC users by age.

	Never EC Users (*n* = 24,663) ^1^				
	<15 Years Old (*n* = 10,988, EC Use Susceptibility = 12.4%)		≥15 Years Old (*n* = 13,675, EC Use Susceptibility = 12.1%)		*p* for Interaction
Favourable Perceptions of ECs Relative to Cigarettes ^2,3^	Crude PR (95% CI)	Adjusted PR ^4^ (95% CI)	Crude PR (95% CI)	Adjusted PR ^4^ (95% CI)	
Less harmful to users	2.78 (2.50–3.09) ***	2.61 (2.34–2.92) ***	2.20 (2.00–2.42) ***	2.00 (1.83–2.19) ***	***
Less harmful to others	2.47 (2.22–2.74) ***	2.33 (2.06–2.64) ***	2.09 (1.90–2.30) ***	1.91 (1.75–2.09) ***	**
More attractive	3.55 (3.11–4.06) ***	3.15 (2.74–3.63) ***	3.05 (2.68–3.47) ***	2.61 (2.27–2.99) ***	*
Easier for minors to buy	2.97 (2.64–3.34) ***	2.80 (2.47–3.17) ***	2.16 (1.93–2.42) ***	1.96 (1.75–2.19) ***	***
More environmentally friendly	2.36 (2.10–2.65) ***	2.20 (1.94–2.51) ***	1.93 (1.74–2.15) ***	1.78 (1.59–1.98) ***	**
More convenient	2.41 (2.14–2.72) ***	2.19 (1.94–2.47) ***	1.98 (1.77–2.21) ***	1.76 (1.60–1.94) ***	**
Cleaner	2.43 (2.18–2.71) ***	2.32 (2.04–2.64) ***	1.96 (1.78–2.17) ***	1.85 (1.68–2.04) ***	**
**Number of favourable perceptions**					
0	1	1	1	1	
1–2	2.53 (2.15–2.99) ***	2.47 (2.04–2.99) ***	2.10 (1.79–2.46) ***	2.01 (1.74–2.32) ***	
3–4	3..89 (3.29–4.60) ***	3.70 (3.04–4.51) ***	3.50 (2.98–4.10) ***	3.18 (2.70–3.75) ***	
5–13	6.68 (5.68–7.86) ***	6.21 (5.10–7.56) ***	5.60 (4.77–6.57) ***	4.77 (4.04–5.63) ***	*
*p* for trend	<0.001	<0.001	<0.001	<0.001	*

^1^ Students not knowing ECs were excluded. ^2^ The reference group for each favourable perception comprised students who did not select the corresponding perception. ^3^ Only favorable perceptions with significant interaction with age are shown. ^4^ Adjusted for sex, perceived family affluence, highest parental education, ever smoking status, family smoking, and school clustering effect. * *p* < 0.05, ** *p* < 0.01, *** *p* < 0.001.

**Table 5:** Association between favourable perceptions of ECs relative to cigarettes and susceptibility in never EC users by sex.

	Never EC Users (*n* = 24,663) ^1^				
	Boys (*n* = 11,826, EC Use Susceptibility = 11.7%)		Girls (*n* = 12,837, EC Use Susceptibility = 12.8%)		*p* for Interaction
Favourable Perceptions of ECs Relative to Cigarettes ^2,3^	Crude PR (95% CI)	Adjusted PR ^4^ (95% CI)	Crude PR (95% CI)	Adjusted PR ^4^ (95% CI)	
More attractive	2.91 (2.50–3.38) ***	2.49 (2.15–2.87) ***	3.53 (3.13–3.98) ***	3.10 (2.66–3.61) ***	*
EC use in children is better accepted by parents	2.65 (2.03–3.48) ***	2.20 (1.78–2.70) ***	3.68 (3.01–4.49) ***	3.15 (2.64–3.77) ***	**
**Number of favourable perceptions**					
0	1	1	1	1	
1–2	1.98 (1.69–2.31) ***	1.94 (1.64–2.29) ***	2.67 (2.25–3.16) ***	2.60 (2.20–3.09) ***	*
3–4	3.27 (2.79–3.83) ***	3.02 (2.52–3.62) ***	4.22 (3.56–5.00) ***	3.98 (3.29–4.83) ***	*
5–13	5.07 (4.32–5.94) ***	4.51 (3.81–5.33) ***	7.35 (6.21–8.69) ***	6.65 (5.37–8.22) ***	**
*p* for trend	<0.001	<0.001	<0.001	<0.001	**

^1^ Students not knowing ECs were excluded. ^2^ The reference group for each favourable perception comprised students who did not select the corresponding perception. ^3^ Only favorable perceptions with significant interaction with sex are shown. ^4^ Adjusted for age, perceived family affluence, highest parental education, ever smoking status, family smoking, and school clustering effect. * *p* < 0.05, ** *p* < 0.01, *** *p* < 0.001.

## Supplementary Materials

The following are available online at www.mdpi.com/1660-4601/15/01/0054/s1, Table S1: Association between EC use susceptibility and ever EC use.
