# Supplementary material for: Favourable Perceptions of Electronic Cigarettes Relative to Cigarettes and the Associations with Susceptibility to Electronic Cigarette Use in Hong Kong Chinese Adolescents
Source: Int J Environ Res Public Health. 2018 Jan 1;15(1):54. doi: 10.3390/ijerph15010054 (PMC5800153; doi:10.3390/ijerph15010054)
Supplement: Supplementary file 1 [file ijerph-15-00054-s001.pdf]

Supplementary File

Table S1. Association between EC use susceptibility and ever EC use (n = 40,202).

EC use susceptibility	Crude PR (95% CI)	Adjusted PR [1] (95% CI)
Not susceptible	1	1
Susceptible	18.25 (16.48-19.78) ***	10.11 (8.70-11.74) ***

[1] Adjusted for age, sex, perceived family affluence, highest parental education, ever smoking status, family smoking and school clustering effect. ***$p < 0.001$.
